# The current state of mental healthcare in Bangladesh: part 2 – setting priorities

**DOI:** 10.1192/bji.2021.42

**Published:** 2021-11

**Authors:** M. Tasdik Hasan, Tasnim Anwar, Enryka Christopher, Sahadat Hossain, Md Mahbub Hossain, Kamrun Nahar Koly, K. M. Saif-Ur-Rahman, Helal Uddin Ahmed, Nazish Arman, Saima Wazed Hossain

**Affiliations:** 1 Consultant (Mental Health), Shuchona Foundation, Dhaka, Bangladesh. Email: tasdik.hasan@liverpool.ac.uk; 2 Graduate Student, Kings College London, UK; 3 Center for Population and Development Studies, Harvard T.H. Chan School of Public Health, Cambridge, MA, USA; 4 Lecturer, Department of Public Health & Informatics, Jahangirnagar University, Savar, Dhaka, Bangladesh; 5 DrPH Researcher, Department of Health Promotion and Community Health Sciences, Texas A&M School of Public Health, College Station, TX, USA; 6 Assistant Scientist, Health Systems and Population Studies Division, icddr,b, Dhaka, Bangladesh; 7 Associate Professor, Child & Adolescent Psychaitry, National Institute of Mental Health, Shere-Bangla Nagar, Dhaka, Bangladesh; 8 Lead Coordinator for Content Development, Shuchona Foundation, Dhaka, Bangladesh; 9 Chairperson, Shuchona Foundation, Dhaka, Bangladesh

**Keywords:** Mental health, mental health system, mental health policy, priorities, Bangladesh

## Abstract

This is the second of a two-part profile on mental healthcare in Bangladesh. It describes the state of mental health research in the country and presents a set of priorities for addressing improvements to the fundamental gaps in mental healthcare highlighted in part 1. Focus on building infrastructure for public mental health facilities, training skilled mental health professionals, adequate distribution of financial resources and addressing stigma are all priorities that will contribute to significantly improving mental healthcare in Bangladesh.

## Background

Part 1 of this two-part country profile gives demographic data on Bangladesh and outlines current mental healthcare services, highlighting the neglect of the significant mental healthcare problem in the country.^1^ There are few public mental health facilities, a scarcity of skilled mental health professionals, insufficient and inequitably distributed financial resources and societal stigma about mental illness. These shortcomings are sustained by the absence of effective stewardship to execute adequate mental health policies. In part 2 we describe the state of mental health research in the country, focus on fundamental gaps in the healthcare system and suggest priorities for addressing improvements.

## State of mental health research in the country

Because mental illness has not been perceived to be a significant health concern in Bangladesh, it has received little research funding. Data on mental disorders from epidemiological studies are therefore limited. A systematic review by Hossain et al demonstrated that research on mental illness was unsatisfactory, given the magnitude of the problem in the country. They mention limitations of their review, including non-comparability of data from selected articles because of differences in settings (clinic versus community), assessment tools and diagnostic thresholds, that further demonstrate the need for more robust research to be conducted on this topic.^2^

The most common screening and diagnostic tools used in Bangladeshi mental health-related studies were the Self-Reporting Questionnaire (SRQ), Patient Health Questionnaire (PHQ) and Structured Clinical Interview for the Diagnostic and Statistical Manual of Mental Disorders (SCID), but some articles published in local journals do not specify the tools used. The earliest preliminary study within a Bangladesh urban setting, conducted in 1975, reported that 31% of out-patients had pure psychogenic conditions.^3^ Hossain et al analysed prevalence estimates from 1975 to 2013 but could not assess the trends over time in the country. Established prevalence rates of psychiatric disorders are prone to underestimation due to social desirability bias of patients and families. Underestimation can also be the result of sufferers being unaware of their mental illness, possibly because of causation beliefs, which prevents proper help-seeking.^2^ Additionally, the differing tools for screening and the various cut-off values used in the reported studies contributed to the various prevalences reported,^4–12^ complicating proper assessment of the situation.

The first national survey conducted, which took place between 2003 and 2005, reported a high burden of mental illness in the country, with approximately 16.1% of adults suffering from at least one type of mental illness.^2,13^ Since then, there has been one more nationwide survey, conducted in 2019, which found a slightly higher prevalence, of 18.7%.^14^ However, the small number of large-scale studies prevents a proper assessment of trends and estimation of the overall need for resources to address the current burden arising from mental illness. Underreporting and underdiagnosis are major challenges for the future of psychiatric epidemiology in Bangladesh.^2^ Further large-scale, well-designed epidemiological studies and clinical trials are needed to generate evidence to improve mental health services in the country.

## Setting priorities for Bangladesh

Our review identified a number of priorities for improving mental healthcare in Bangladesh. These are summarised in Box 1 and expanded on in this section.

BOX 1 Priorities for improving mental healthcare in Bangladesh

Research

- Estimate the disease burden for mental illness
- Secure funding, design and implement cost-effective interventions to manage and prevent psychiatric disorders
- Execute effective data management, monitoring and evaluation strategies

Service development

- Implement mhGAP at the national level
- Integrate mental healthcare at the primary care level
- Develop new human resources and improve existing human resources

Advocacy

- Educate the public about mental health
- Reduce stigma and discrimination by health care providers
- Encourage young advocates to become involved

### Research priorities

A major challenge encountered while preparing this country profile included sorting old and inconsistent data on mental disorders in Bangladesh. The prevalence of psychiatric disorders is likely underestimated owing to incomparable data between studies, bias in reporting and stigma. Large-scale epidemiological studies are needed to update national statistics on mental disorders, standardise diagnostic tools, estimate incidence rates and the burden of psychiatric illness more accurately, and quantify impacts in globalised terms such as disability-adjusted life-years (DALYs). With treatment gaps as large as 92% for adults and 94% for children according to some studies,^15^ reducing the burden of mental illness should be the ultimate goal of the government health ministry.

Designing and implementing sustainable cost-effective interventions by conducting operational research on prevention and treatment of mental disorders should be a research priority of the country. Implementation must be accompanied by ongoing research to examine feasibility, applicability and sustainability. Vulnerability should also be addressed during the design of these studies.

Expansion of research in five essential domains – epidemiology, effects of violence, women's mental health, prevention and mental health services (including digital approaches) – should be prioritised because of the demonstrated need in these areas. Data collection and monitoring mechanisms must be strengthened. The international community must begin to help make mental health a priority for Bangladesh by contributing substantial resources.

### Service development priorities

The World Health Organization (WHO) Mental Health Gap Action Programme (mhGAP) is prepared to scale up services for mental, neurological and substance use disorders, especially in low- and middle-income countries. However, the mhGAP initiative is yet to be implemented in Bangladesh, with the exception of a population of forcibly displaced Myanmar nationals/Rohingya living in refugee camps in the south-east region of the country.^15^ Nationwide implementation of the mhGAP, integration of the treatment of psychiatric disorders into primary care settings, provision of training, overseeing the practice of primary care staff, adequate supervision and increased referral capacity of secondary care centres would greatly improve the provision of mental health services as a whole. The training required to decrease the mental health treatment gap at the secondary level of care would only be 85 days for district-level doctors and 28 days for county/borough-level doctors.^15^ Bangladesh has a large number of community health workers who can be trained to deliver psychosocial support to their wider communities, which would be a cost-effective means of greatly supporting mental healthcare. At the ward level, there is at least one community clinic for approximately 6000 people. The union and subdistrict facilities work for community clinic services. The Directorate General of Family Planning has almost 13 500 full-time community healthcare providers for the community clinic.^16^ These healthcare workers should be trained to allow for the integration of mental health services into the existing primary healthcare system.

Mental health education should be re-evaluated, and a new model of healthcare that incorporates the biopsychosocial model of care should be developed and taught in healthcare education programmes. This will build capacity by enabling future healthcare professionals to gain leadership positions and improve training programmes. Operational research should be the priority for mental health service development.

### Advocacy priorities

While analysing the literature for this country profile, we have seen the widespread stigma surrounding mental illness prevalent across the country. Therefore, a strong advocacy plan is needed to educate the public through utilisation of mass media, setting-based approaches and peer education. The population should be educated about causation, which can be done through healthcare promotion activities in communities and through mass media.^2,17,18^ Public and professional awareness must increase in order to reduce stigma about mental disorders and discrimination among patients. Young advocates, including academics, media personalities, professionals, service providers and policymakers, can play important roles in advocacy.

## Conclusions

Much improvement is required in the mental health sector of Bangladesh, including within the country's socio-cultural context, existing policies and services. Investing funds into researching effective interventions, integrating mental healthcare at the primary level and educating the public about mental health and stigma are among the main priorities in improving mental health in Bangladesh.

## Data availability

Data availability is not applicable to this article as no new data were created or analysed in this study.
